# Exploring information technology utilization and needs in community pharmacies: a cross-sectional survey in Shanghai, China

**DOI:** 10.3389/fphar.2025.1554141

**Published:** 2025-02-11

**Authors:** Qin Li, Zhao Jin, Yun Liao, Huihua Dai, Jie Meng, Ling Li

**Affiliations:** ^1^ Department of Pharmacy, Tongren Hospital, Shanghai Jiao Tong University School of Medicine, Shanghai, China; ^2^ Department of Pharmacy, Community Health Service Center of Xinjing Town, Shanghai, China

**Keywords:** community pharmacist, information technology, pharmaceutical care, China, survey

## Abstract

**Background:**

Community pharmacists are critical in managing medication therapy and ensuring patient safety. However, in many countries, including China, challenges such as limited access to real-time patient data, fragmented communication systems, and underdeveloped clinical decision-support tools hinder the effectiveness of pharmacy services. Integrating information technology (IT) into community pharmacies can improve medication management by providing timely access to patient information, enhancing medication safety, and facilitating communication with healthcare providers.

**Objective:**

This study aimed to explore the medication management practices of community pharmacists, the existing IT support available, and their specific requirements for IT-assisted medication management in community pharmacies in Shanghai, China.

**Methods:**

An online self-assessment survey was conducted to evaluate the utilization and needs of IT-based medication management among community pharmacists in Shanghai. Demographic data and information on pharmaceutical care practices were collected and analyzed to examine correlations between these factors and the need for IT support.

**Results:**

963 out of 2,178 surveys were completed over 6 months. Nearly all respondents (99.3%) provided at least one type of pharmaceutical care, but only 0.93% offered all types. Regarding current utilization and needs for IT systems, community pharmacists rated access to patient/medication information and digital standardized documentation flow as poor satisfaction with the highest level of needs. Multivariate regression analysis showed that IT needs were significantly associated with time spent in clinical pharmacy practice, time dedicated exclusively to clinical pharmacy, and the number of prescriptions dispensed weekly.

**Conclusion:**

While community pharmacists are key in providing pharmaceutical care, IT support remains inadequate. There is a pressing need for an integrated IT system that offers real-time access to patient medical information, a standardized documentation system, improved clinical decision support software, and enhanced communication with other healthcare providers.

## 1 Introduction

The incidence and burden of chronic diseases are increasing globally (Global Burden of Disease Study, 2013 Collaborators, 2015). Although appropriate drug treatment is associated with improved clinical outcomes and prognosis, medication use is often accompanied by issues that lead to poor health outcomes and increased healthcare costs (Freitas et al., 2017). Recent studies suggest that many of these issues are medication therapy problems (MTPs) related to the use of medications across diverse populations rather than inappropriate prescribing (Steinman et al., 2011). Community pharmacists, who manage medication use and identify and resolve MTPs, have become key in optimizing medication management worldwide (Mumbi et al., 2024; Witry and Doucette, 2014).

The role of information technology (IT)-mediated medication management in supporting community pharmacists to ensure patient safety, enhance treatment efficiency, and improve the quality of care has been emphasized for decades. IT-based medication management has evolved in various forms, including Prescription Drug Monitoring Programs (PDMPs) in the United States (Tay et al., 2023), PharmaNet in the Province of British Columbia, Canada (Dormuth et al., 2012), e-Medikation cloud medication records in three pilot regions of Austria (Ammenwerth et al., 2014), the Cloud Emergency Medical Service System in India (Rajkumar and Sriman Narayana Iyengar, 2013), and the NHI-Pharma Cloud in Taiwan (Huang et al., 2015). Studies have shown that integrating IT-based systems into practice allows community pharmacists to access timely, comprehensive medication information, facilitating problem-solving, decision-making, and patient education and improving patient safety and drug therapy outcomes, particularly for patients with complex or chronic conditions (Chen et al., 2016; Fanizza et al., 2018).

China, with the largest aging population in the world, is experiencing rapid demographic changes and an increasing prevalence of multimorbidity (defined as two or more coexisting chronic conditions) (Chen et al., 2022). The aging population and increasingly complex medication regimens have escalated the demand for pharmacy services (Sikka et al., 2015). Without IT-mediated medication management systems, pharmacists’ workflow efficiency is hindered, limiting their ability to identify and resolve MTPs (van Stiphout et al., 2015). In 2017, the National Health Commission formulated the Action Plan to further improve medical services, which emphasized that pharmacists should use information technology to provide personalized advice for rational drug consumption to outpatients and inpatients and to provide pharmaceutical services up to primary care. In 2018, the China National Health Commission and the State Administration of Traditional Chinese Medicine jointly issued the “Opinions on Accelerating the High-Quality Development of Pharmaceutical Care”, which advocates enhancing pharmaceutical care capabilities through IT-based medication management. At present, community pharmacy in China is still in its infancy and information development has just begun. To optimize and improve community pharmaceutical services, it is important to leverage digital capabilities to build an intelligent, collaborative pharmaceutical service system.

Despite these advancements, key questions remain: What is the current level of Chinese community pharmacists’ involvement in pharmaceutical care? To what extent does IT support help pharmacists optimize drug supply? What aspects of IT-mediated medication management are most needed by community pharmacists? While some studies have examined the IT support needs of hospital pharmacists in China (Wang et al., 2020), studies have yet to specifically focus on the use and needs of IT in community pharmacy settings. This study aims to assess the status of community pharmacists’ involvement in medication management, evaluate the IT support available to them, and identify their needs for IT-mediated medication management using a self-assessment questionnaire.

## 2 Material and methods

### 2.1 Study design and participants

This cross-sectional study used a self-administered online questionnaire distributed via the WeChat platform (the largest social media in China) through online links. The survey was conducted among all full-time pharmacists working in community pharmacies in Shanghai from March to September 2023. Licensed pharmacists were invited to voluntarily respond to the questionnaire. Pharmacists who worked part-time as substitutes in administrative or wholesale departments or community pharmacies located within hospital premises were excluded based on a screening question at the beginning of the survey.

### 2.2 Sample size calculation and questionnaire development

The sample size was determined using the Raosoft^®^ sample size calculator (http://www.raosoft.com/samplesize.html) with a 95% confidence level, a 5% margin of error, and a study population size of 2,178. Assuming a 50% prevalence of pharmaceutical care, the calculated sample size was 327.

Using a mixed model Q methodology (Valenta and Wigger, 1997), the questionnaire was developed based on the technical specifications of the service project in China and the five practice principles for pharmaceutical care developed by the American Pharmacist Association (APhA),previous information needs research strategies (McPherson et al., 2020; Wang et al., 2020), and the core competencies for entry-level professionals proposed by the Drug Administration Office of the Shanghai Health Commission.

### 2.3 Evaluation of community pharmacist’ services and IT system

The evaluation of the current services provided by community pharmacists was based on the five practice principles of pharmaceutical care outlined by APhA. (1) Data collection, (2) Information evaluation, (3) Formulating a plan, (4) Implementing the plan, and (5) Monitoring and modifying the plan.

The evaluation of community pharmacists’ utilization of and needs for IT systems was based on key information technologies that support pharmacy practice (Ammenwerth et al., 2014; Dormuth et al., 2012; Huang et al., 2015; Rajkumar and Sriman Narayana Iyengar, 2013; Tay et al., 2023; Witry and Doucette, 2014), including: (1) Accessing patient medical and medication information, (2) Clinical decision support software, (3) Communication with other healthcare providers, and (4) Digital standardized documentation workflows.

### 2.4 Data collection

The questionnaire was completed on the WeChat platform through online links. To prevent duplicate submissions, each respondent was allowed to complete the survey on only one electronic device (mobile phone or computer).

### 2.5 Data analysis

All data were entered and analyzed using the Statistical Package for the Social Sciences (SPSS) version 25.0 (IBM Corp., Armonk, New York). Descriptive statistics were calculated for all variables. The association between variables was assessed using univariate Spearman correlation analysis. Variables with a p-value ≤ 0.20 were analyzed using multiple logistic regression under the generalized linear model function. Results with a p-value < 0.05 were considered statistically significant.

Community pharmacists’ level of satisfaction with and need for IT-mediated medication management was assessed using a 6-point Likert-like scale (Table 1). The scale ranged from 5 (Extremely Satisfied/Highest needs) to 0 (Extremely Dissatisfied/No need), with intermediate categories as follows: 4 (Very Satisfied/High needs), 3 (Satisfied/Moderate needs), 2 (Below Average Satisfied/Low needs), and 1 (Poorly Satisfied/Lowest needs). The average and standard deviation were calculated for each item. The scoring range used to interpret the means (Table 2) was calculated using the following formula (35): Range of score = (Maximum score − Minimum score)/6 = (5–0)/6 = 0.83.

**TABLE 1:** Levels of community pharmacists’ satisfaction with current IT support and needs for IT-mediated medication management.

Score	Satisfaction with current IT support	Needs for IT support
5	Extremely satisfied	Highest needs
4	Very satisfied	High needs
3	Satisfied	Moderate needs
2	Below average satisfaction	Low needs
1	Poorly satisfied	Lowest needs
0	Extremely unsatisfied	No need

**TABLE 2:** Range of score and interpretation.

Score range	Satisfaction with current IT system	IT support needs
4.16–5.00	Extremely satisfied	Highest needs
3.33–4.15	Very satisfied	High needs
2.50–3.32	Good satisfied	Moderate needs
1.67–2.49	Below Average satisfaction	Low needs
0.84–1.66	Poorly satisfied	Lowest needs
0.00–0.83	Extremely unsatisfied	No need

## 3 Results

### 3.1 Survey response and demographic characteristics

A total of 2,178 questionnaires were distributed to 248 community pharmacies across Shanghai (the largest city and one of the first cities to promote community medicine supply in China, often chosen by the Chinese central government as a model city for implementing new policy reforms), and 963 (44.2%) were returned, meeting the required sample size of 327. The respondents were primarily female (75.3%), the majority between the ages of 31 and 50 (65.5%). In terms of education, 63.2% held a bachelor’s or master’s degree in pharmacy, and over half (51.2%) had completed standardized clinical pharmacist training in Shanghai. Regarding professional experience, 39.2% had 6–10 years in clinical pharmacy practice, and 41.4% dedicated 5–10 h per week to clinical tasks. Regarding workload, 57.0% conducted 3-7 clinical services per week, and 45.9% dispensed between 1,000 and 2,000 prescriptions per week. Details are shown in Table 3.

**TABLE 3:** Frequency and percentage of respondents classified by demographic details (n = 963).

Participants’ demographics	Options to select	N (%)
Gender	Male	238 (24.7%)
	Female	725 (75.3%)
Age	20–30 years	189 (19.6%)
	31–40 years	289 (30.0%)
	41–50 years	342 (35.5%)
	51–60 years	127 (13.2%)
	Over 60 years	16 (1.7%)
Highest level of education	Junior college degree	354 (36.8%)
	Bachelor’s degree	589 (61.2%)
	Master’s degree	20 (2.0%)
Finished standardized training	Yes	493 (51.2%)
	No	470 (48.8%)
Time in clinical pharmacy practice	<1 year	124 (12.9%)
	1–5 years	154 (16.0%)
	6–10 years	378 (39.2%)
	11–20 years	279 (29.0%)
	>20 years	28 (2.9%)
Hours/week in clinical pharmacy	<5 h	114 (11.8%)
	5–10 h	399 (41.4%)
	11–15 h	222 (23.1%)
	16–20 h	171 (17.8%)
	>20 h	55 (5.9%)
Average number of clinical services	<2	79 (8.2%)
	3–7	549 (57.0%)
	8–12	304 (31.6%)
	>13	31 (3.2%)
Number of prescriptions dispensed	0–100	27 (2.8%)
	101–500	112 (11.6%)
	501–1,000	344 (35.8%)
	1,001–2,000	442 (45.9%)
	>2,000	38 (3.9%)

### 3.2 Types of pharmacy services provided

Table 4 summarizes the practice of the five core principles of APhA pharmaceutical care among the respondents. Nearly all pharmacists (99.3%) ensured that patients understood the purpose of their medications. Additionally, 84.6% of respondents ensured that patients understood their current health status, which is vital for informed decision-making regarding treatment options. Another typical service was gathering health and medical history from patients or their caregivers, reported by 82.6% of pharmacists. However, the comprehensive application of the five APhA practice principles was less common. Only 0.93% (9) of respondents provided all components of these principles.

**TABLE 4:** Practice of the five core principles of pharmaceutical care among respondents (n = 963).

Pharmaceutical care principle	Description	N (%)
Ensuring patients understand the purpose of their medications	Provide education on medication purpose	956 (99.3%)
Ensuring patients understand their current health status	Explain current health status to patients	815 (84.6%)
Gathering health and medical history from patients/caregivers	Collect medical histories	795 (82.6%)
Data collection	Collect relevant data for medication management	87 (9.0%)
Information evaluation	Evaluate collected data for potential issues	62 (6.4%)
Formulating a care plan	Develop a personalized care plan based evaluation	36 (3.7%)
Implementing the care plan	Apply the care plan with patient involvement	14 (1.4%)
Monitoring and modifying the care plan	Adjust care plan based on patient progress	9 (0.9%)

### 3.3 Community pharmacists’ evaluation of the current IT system

Table 5 evaluates the current information systems used by community pharmacists. Overall, respondents expressed dissatisfaction with various components of the existing IT infrastructure. The lowest satisfaction was reported for “access to patient medical and medication information”, with an average score of 0.97 ± 0.72, indicating “poor satisfaction”. Similarly, “clinical decision support software” (1.71 ± 0.81), “communication with other healthcare providers” (1.51 ± 0.83), and “digital standardized documentation workflow” (1.05 ± 0.69) all received low satisfaction ratings. This dissatisfaction highlights the urgent need for improvements in these key areas to enhance pharmacists’ ability to manage patient care effectively.

**TABLE 5:** Level of satisfaction with the current information system by community pharmacists (n = 963).

Current information system	5	4	3	2	1	0	Mean ± S.D.
Access to patient’s medical and medication information	1	7	32	85	634	204	0.97 ± 0.72
Clinical decision support software	2	9	81	581	194	96	1.71 ± 0.81
Communication with other patients’ healthcare providers	6	28	41	353	483	52	1.51 ± 0.83
Digital Standardized documentation workflow	0	4	12	179	598	170	1.05 ± 0.69

### 3.4 Assessment of necessity for IT-mediated medication management

The survey also assessed the perceived necessity for IT-mediated medication management, as shown in Table 6. Respondents rated “access to patient medical and medication information” (4.17 ± 1.17) and “digital standardized documentation workflow” (4.17 ± 1.09) as having the highest needs. In contrast, “clinical decision support software” (2.79 ± 1.31) and “communication with other healthcare providers” (2.91 ± 1.36) were rated as moderate needs. This indicates a strong preference for improvements in patient data access and documentation workflows over clinical decision support and communication tools.

**TABLE 6:** Necessity for IT-Mediated medication management by community pharmacists (n = 963).

Needs for information system	5	4	3	2	1	0	Mean ± S.D.
Access to patient’s medical and medication information	518	261	68	64	48	4	4.17 ± 1.17
Clinical decision support software	118	176	223	319	84	43	2.79 ± 1.31
Communication with other patients’ healthcare providers	127	239	232	189	143	33	2.91 ± 1.36
Digital Standardized documentation workflow	486	289	86	72	24	6	4.17 ± 1.09

### 3.5 Factors associated with the need for IT-mediated medication management

Eight potential factors were analyzed for their relationship with the need for IT-mediated medication management, including gender, age group, education level, training as a clinical community pharmacist, years in clinical pharmacy practice, number of hours per week dedicated exclusively to clinical pharmacy, average number of clinical services conducted per week, and number of prescriptions dispensed per week. Only factors that showed a significant relationship with IT needs are listed in Table 7.

**TABLE 7:** Factors associated with the necessity for IT-medication management.

Highest/high needs for information system	Factors	Characteristics	Odds ratio (95% CI)	P-value
Access the patients’ medical and medication information	Numbers of hours/week dedicated exclusively to clinical pharmacy (h)	≤10	As reference	
		>10	4.272 (2.444, 7.466)	0.01
	Average number of clinical services conducted a week (n)	≤7	As reference	
		>7	8.189 (4.039, 16.61)	0.01
Clinical decision support software	Time in clinical pharmacy practice	≤10 years	As references	
		>10 years	0.535 (0.348, 0.824)	0.01
Communication with other patients’ healthcare providers	Numbers of hours/week dedicated exclusively to clinical pharmacy (h)	≤10	As reference	
		>10	1.616 (1.131, 2.309)	0.01
	Average number of clinical services conducted a week (n)	<7	As reference	
		>7	1.545 (1.144, 2.087)	0.02
Digital standardized documentation workflow	Number of prescriptions dispensed per week (n)	≤1,000	As reference	
		>1,000	2.112 (1.503, 2.968)	0.01

Key factors associated with higher IT needs included the number of hours dedicated to clinical pharmacy practice, the average number of clinical services conducted per week, and the number of prescriptions dispensed weekly. Specifically, pharmacists who dedicated more than 10 h per week to clinical pharmacy practice and those who conducted more than 7 clinical services per week were significantly more likely to report higher IT needs for accessing patient medical and medication information and communicating with other healthcare providers. Pharmacists who dispensed more than 1,000 prescriptions per week were found to have a greater need for digital standardized documentation workflows. These findings suggest that pharmacists with higher clinical workloads are more likely to identify the necessity for enhanced IT support to improve workflow efficiency and patient care.

## 4 Discussion

This study found that nearly all respondents (99.3%) provided some form of medication care, likely due to increased awareness and demand driven by the National Health Commission of China. However, several areas need to be improved in the support provided by IT systems currently in place to better support pharmacists in their roles.

### 4.1 IT-medication management capability needs improvement

One key finding is that community pharmacists need improvement in the IT systems, especially in providing easy and real-time access to patient medical information. The potential positive impact of drug therapy management on patient care depends heavily on a pharmacist’s ability to recognize and address MTPs. Without timely access to relevant medical records, it becomes more difficult for pharmacists to identify and address potential MTPs. Real-time access to prescription data, laboratory results, and diagnoses can significantly enhance pharmacists’ ability to make informed clinical decisions, improving the quality of patient care and expanding the scope of pharmacy services. Such systems enable pharmacists to make better recommendations and contribute to improved patient outcomes (Hettinger et al., 2024). Although some hospitals in China have real-time patient medication monitoring systems, these systems often focus more on statistical analysis of drug use at the population level rather than providing real-time monitoring of individual patients (Wang et al., 2020).

The study found that over three-fourths of respondents had limited access to patient medical information from other healthcare providers, a challenge like that faced in developing countries (Loh et al., 2021). Respondents rated their satisfaction with the current system as “poor”. They expressed the highest need for improvement, particularly among those who dedicated more than 10 h per week to clinical pharmacy or performed more than seven clinical services per week. The greater the hours and services, the greater the need for comprehensive access to patient information. The increasing attention the Chinese government gave to developing modern informatics platforms that integrate patient information could help address this issue and support community pharmacists in accessing comprehensive medical and medication data for patients (Xu, 2024). Integrating an IT system that allows community pharmacists to access and securely share a patient’s medical information, including diagnoses, care plans and laboratory results, is essential.

### 4.2 Clinical decision support software needs enhancement

Clinical decision support systems (CDSS) are vital for improving patient care by offering real-time alerts and reminders during medication delivery. Integrating CDSS into community pharmacy practice can optimize workflows, improve patient counseling, reduce drug interactions, and enhance medication safety (Curtain and Peterson, 2014). In China, CDSS has been increasingly implemented to support physicians and pharmacists in primary care, particularly in areas like anticoagulation management (Ru et al., 2023) and anti-infective treatment (Wang et al., 2020).

However, respondents in this study rated the current clinical decision support software as “below average” in terms of satisfaction, with a moderate need for improvement. A significant limitation noted by respondents was the excessive number of irrelevant alerts generated by the software, which requires pharmacists to investigate these irrelevant warnings, leading to frustration and dissatisfaction. This aligns with findings from other studies, which indicate that pharmacists often prefer digital formats, especially those with easy access to a free, user-friendly database, over systems that bombard them with irrelevant information (Jin et al., 2024). Interestingly, less experienced community pharmacists (with less than 10 years of clinical experience) reported a greater need for CDSS, highlighting the opportunity for improved, tailored decision-support tools for community pharmacists. We recommend involving community pharmacists in the development and management of CDSS to limit alert fatigue, make the system more efficient, and tailor it to pharmacists’ specific needs.

### 4.3 Communication with other healthcare providers is inefficient

As community pharmacy practice evolves from medication dispensing to a more collaborative and patient-centered approach, building inter-professional relationships is crucial to ensuring high-quality patient care (Manolakis and Skelton, 2010). Barriers to communication between pharmacists and other healthcare providers, such as physicians, present challenges in managing patients with chronic diseases, as noted in previous studies in China (Cai et al., 2023).

In this survey, communication channels between physicians and pharmacists were rated as “poorly satisfied”, with a moderate need for improvement. This issue may stem from community pharmacists often communicating directly with patients rather than healthcare providers (Cai et al., 2023). However, the need for improved communication channels was greater among those who provided more than 7 clinical services per week and dedicated more than 10 h per week to clinical pharmacy, possibly because a higher volume/time of services increases the likelihood of needing to discuss treatment plans with physicians. To address these communication barriers, effective interventions should be developed to promote stronger interprofessional relationships and create a conducive communication platform.

### 4.4 Standardized documentation systems need improvement

Community pharmacists must document their clinical services to track patient outcomes and provide evidence of service quality. Emerging technologies, including web-based medication management applications, are being developed to help pharmacies integrate clinical data, document services, and monitor patient outcomes (Turner et al., 2018).

However, despite the importance of documentation, many respondents who need to process large amounts of health information manually chose not to document due to a lack of interoperable documentation systems. Such inefficiencies may lead to incomplete documentation, which can impact workflow and patient care quality (Smith et al., 2017; Turner et al., 2018).

Respondents rated the current digital standardized documentation workflow as “poorly satisfied”, with a strong need for improvement, particularly for those dispensing more than 1,000 prescriptions per week. To address these barriers, there is an urgent need for more efficient and user-friendly digital documentation systems that enable clinical performance documentation, clinical data integration, and patient outcome tracking to better support pharmacy management.

### 4.5 Recommendations to IT-support system to community pharmacists

The role of community pharmacists in clinical care is increasing in China, and more support is needed to implement IT systems beyond the dispensing workflow. It is possible that some IT strategies are most needed in the implementation process, while others are needed later for sustained improvement of the IT system. Additionally, some strategies may only be required during one implementation phase, while others may be required across multiple phases. For example, the most urgent and diverse processes that pose IT requirements are the provision of easy, real-time access to patient medical information and a digital, standardized documentation workflow. Different types of IT strategies may be more effective at certain pharmacies, depending on the community pharmacist’s work content, clinical drug supply practice, e.g., Clinical decision support software is in greater need among less experienced community pharmacists.

Currently, structural barriers limit community pharmacists’ access to patient medical information, including incompatible information systems between different medical institutions and lack of authority for community pharmacists to use the patient medical information system. As the Chinese government pays increasing attention to modern informatics platforms that integrate patient information, regular follow-up surveys are recommended to test these policy changes and technological advances and ensure that IT systems continue to meet pharmacists’ needs. In addition, concrete case analyzes are proposed to demonstrate the concrete possible uses of IT systems in community pharmacies and to show how these systems solve practical problems and thus increase the practicality and persuasiveness of the study.

### 4.6 Limitations of the study

This study has several limitations. First, not all community pharmacists in Shanghai completed the online self-administered questionnaire, and there may be differences in IT support needs between respondents and non-respondents. However, the response rate of 44.2% is relatively high, and the demographic distribution of respondents closely mirrors that of community pharmacies across the regions of Shanghai. Second, this study is the first in China to assess pharmaceutical care based on the five principles established by the APhA. While the principles used to evaluate carefully include the main content of current community pharmaceutical services in China, some of the components used to verify these principles may not be foolproof. Nevertheless, respondents reported no difficulty understanding the questionnaire, suggesting that the five principles are an appropriate framework for assessing current community pharmacy practice in China.

## 5 Conclusion

Most participating community pharmacists are engaged in at least some form of pharmaceutical care, but the support provided by information systems requires significant improvement. There is an urgent need to develop a pharmacist-friendly, integrated system that allows real-time access to patient medical information and a standardized documentation workflow. Improvements are also needed in communication capabilities between physicians and pharmacists and in the ability of IT medication software to effectively support clinical decision-making.

## Data Availability

The original contributions presented in the study are included in the article/supplementary material, further inquiries can be directed to the corresponding authors.

## References

[B1] Ammenwerth E. Duftschmid G. Gall W. Hackl W. O. Hoerbst A. Janzek-Hawlat S. (2014). A nationwide computerized patient medication history: evaluation of the Austrian pilot project e-Medikation. Int. J. Med. Inf. 83 (9), 655–669. 10.1016/j.ijmedinf.2014.06.004 24986321

[B2] Cai S. Huang X. Van C. Li W. Yan M. Lu Y. (2023). General practitioners' attitudes towards and frequency of collaboration with pharmacists in China: a cross-sectional study. BMC Health Serv. Res. 23 (1), 1174. 10.1186/s12913-023-10151-0 37891601 PMC10612245

[B3] Chen C. M. Kuo L. N. Cheng K. J. Shen W. C. Bai K. J. Wang C. C. (2016). The effect of medication therapy management service combined with a national PharmaCloud system for polypharmacy patients. Comput. Methods Programs Biomed. 134, 109–119. 10.1016/j.cmpb.2016.07.008 27480736

[B4] Chen X. Giles J. Yao Y. Yip W. Meng Q. Berkman L. (2022). The path to healthy ageing in China: a Peking University-Lancet Commission. Lancet 400 (10367), 1967–2006. 10.1016/S0140-6736(22)01546-X 36423650 PMC9801271

[B5] Curtain C. Peterson G. M. (2014). Review of computerized clinical decision support in community pharmacy. J. Clin. Pharm. Ther. 39 (4), 343–348. 10.1111/jcpt.12168 24806361

[B6] Dormuth C. R. Miller T. A. Huang A. Mamdani M. M. Juurlink D. N. Canadian Drug S. (2012). Effect of a centralized prescription network on inappropriate prescriptions for opioid analgesics and benzodiazepines. CMAJ 184 (16), E852–E856. 10.1503/cmaj.120465 22949563 PMC3494359

[B7] Fanizza F. A. Ruisinger J. F. Prohaska E. S. Melton B. L. (2018). Integrating a health information exchange into a community pharmacy transitions of care service. J. Am. Pharm. Assoc. 58 (4), 442–449. 10.1016/j.japh.2018.02.012 29625912

[B8] Freitas G. R. M. Tramontina M. Y. Balbinotto G. Hughes D. A. Heineck I. (2017). Economic impact of emergency visits due to drug-related morbidity on a Brazilian hospital. Value Health Reg. Issues 14, 1–8. 10.1016/j.vhri.2017.03.003 29254532

[B9] Global Burden of Disease Study 2013 Collaborators (2015). Global, regional, and national incidence, prevalence, and years lived with disability for 301 acute and chronic diseases and injuries in 188 countries, 1990-2013: a systematic analysis for the Global Burden of Disease Study 2013. Lancet 386 (9995), 743–800. 10.1016/S0140-6736(15)60692-4 26063472 PMC4561509

[B10] Hettinger K. N. Adeoye-Olatunde O. A. Russ-Jara A. L. Riley E. G. Kepley K. L. Snyder M. E. (2024). Preparing community pharmacy teams for health information exchange (HIE). J. Am. Pharm. Assoc. (2003) 64 (2), 429–436.e2. 10.1016/j.japh.2023.12.003 38081515

[B11] Huang S. K. Wang P. J. Tseng W. F. Syu F. K. Lee M. C. Shih R. L. (2015). NHI-PharmaCloud in Taiwan--A preliminary evaluation using the RE-AIM framework and lessons learned. Int. J. Med. Inf. 84 (10), 817–825. 10.1016/j.ijmedinf.2015.06.001 26113462

[B12] Jin L. Fang H. Shen J. He Z. Li Y. Dong L. (2024). Evaluation of appropriateness of alerts overrides and physicians' responses of the medication-related clinical decision support system in China, a hospital-based study. Drug Discov. Ther. 18 (2), 89–97. 10.5582/ddt.2024.01012 38658357

[B13] Loh P. Chua S. S. Karuppannan M. (2021). The extent and barriers in providing pharmaceutical care services by community pharmacists in Malaysia: a cross-sectional study. BMC Health Serv. Res. 21 (1), 822. 10.1186/s12913-021-06820-7 34399749 PMC8365940

[B14] Manolakis P. G. Skelton J. B. (2010). Pharmacists' contributions to primary care in the United States collaborating to address unmet patient care needs: the emerging role for pharmacists to address the shortage of primary care providers. Am. J. Pharm. Educ. 74 (10), S7. 10.5688/aj7410s7 21436916 PMC3058447

[B15] McPherson K. L. Adeoye-Olatunde O. A. Osborne J. M. Doucette W. R. Gernant S. A. Jaynes S. (2020). Community pharmacists and residents decision making and unmet information needs when completing comprehensive medication reviews. J. Am. Pharm. Assoc. (2003) 60 (3S), S41-S50.e2–S50 e42. 10.1016/j.japh.2019.12.009 31987810 PMC7299788

[B16] Mumbi A. Mugo P. Barasa E. Abiiro G. A. Nzinga J. (2024). Factors influencing the uptake of public health interventions delivery by community pharmacists: a systematic review of global evidence. PLoS One 19 (8), e0298713. 10.1371/journal.pone.0298713 39088540 PMC11293714

[B17] Rajkumar R. Sriman Narayana Iyengar N. C. (2013). Dynamic integration of mobile JXTA with cloud computing for emergency rural public health care. Osong Public Health Res. Perspect. 4 (5), 255–264. 10.1016/j.phrp.2013.09.004 24298441 PMC3845228

[B18] Ru X. Wang T. Zhu L. Ma Y. Qian L. Sun H. (2023). Using a clinical decision support system to improve anticoagulation in patients with nonvalve atrial fibrillation in China's primary care settings: a feasibility study. Int. J. Clin. Pract. 2023, 2136922. 10.1155/2023/2136922 36713952 PMC9876694

[B19] Sikka R. Morath J. M. Leape L. (2015). The Quadruple Aim: care, health, cost and meaning in work. BMJ Qual. Saf. 24 (10), 608–610. 10.1136/bmjqs-2015-004160 26038586

[B20] Smith M. G. Ferreri S. P. Brown P. Wines K. Shea C. M. Pfeiffenberger T. M. (2017). Implementing an integrated care management program in community pharmacies: a focus on medication management services. J. Am. Pharm. Assoc. (2003) 57 (2), 229–235. 10.1016/j.japh.2016.12.074 28173993

[B21] Steinman M. A. Handler S. M. Gurwitz J. H. Schiff G. D. Covinsky K. E. (2011). Beyond the prescription: medication monitoring and adverse drug events in older adults. J. Am. Geriatr. Soc. 59 (8), 1513–1520. 10.1111/j.1532-5415.2011.03500.x 21797831 PMC3940362

[B22] Tay E. Makeham M. Laba T. L. Baysari M. (2023). Prescription drug monitoring programs evaluation: a systematic review of reviews. Drug Alcohol Depend. 247, 109887. 10.1016/j.drugalcdep.2023.109887 37126936

[B23] Turner K. Renfro C. Ferreri S. Roberts K. Pfeiffenberger T. Shea C. M. (2018). Supporting community pharmacies with implementation of a web-based medication management application. Appl. Clin. Inf. 9 (2), 391–402. 10.1055/s-0038-1651488 PMC5976494 29847843

[B24] Valenta A. L. Wigger U. (1997). Q-methodology: definition and application in health care informatics. J. Am. Med. Inf. Assoc. 4, 501–510. 10.1136/jamia.1997.0040501 PMC61268 9391937

[B25] van Stiphout F. Zwart-van Rijkom J. E. Maggio L. A. Aarts J. E. Bates D. W. van G. (2015). Task analysis of information technology-mediated medication management in outpatient care. Br. J. Clin. Pharmacol. 80 (3), 415–424. 10.1111/bcp.12625 25753467 PMC4574827

[B26] Wang Y. Dai Y. Yang J. Zhou H. Chen Z. Li G. (2020). A survey of Chinese pharmacists participating in anti-infective therapy and its related information technology support. J. Clin. Pharm. Ther. 45 (4), 707–714. 10.1111/jcpt.13152 32403187

[B27] Witry M. J. Doucette W. R. (2014). Community pharmacists, medication monitoring, and the routine nature of refills: a qualitative study. J. Am. Pharm. Assoc. 54 (6), 594–603. 10.1331/JAPhA.2014.14065 25322259

[B28] Xu J. (2024). The current status and promotional strategies for cloud migration of hospital information systems in China: strengths, weaknesses, opportunities, and threats analysis. JMIR Med. Inf. 12, e52080. 10.2196/52080 PMC10877487 38315519

